# Supplementary material for: Iterative improvement in the automatic modular design of robot swarms
Source: PeerJ Comput Sci. 2020 Dec 7;6:e322. doi: 10.7717/peerj-cs.322 (PMC7924708; doi:10.7717/peerj-cs.322)
Supplement: Supplemental Information 3 [file peerj-cs-06-322-s003.zip › argos3/doc/api/standalone/a00637_source.html]

ARGoS: plugins/simulator/visualizations/qt-opengl/qtopengl\_camera.cpp Source File


- Main Page
- Related Pages
- Namespaces
- Classes
- Files

- File List
- File Members

# plugins/simulator/visualizations/qt-opengl/qtopengl\_camera.cpp

Go to the documentation of this file.

```
00001 
00007 #include "qtopengl_camera.h"
00008 #include <QPoint>
00009 #include <argos3/core/utility/math/quaternion.h>
00010 #include <argos3/core/utility/logging/argos_log.h>
00011 
00012 namespace argos {
00013 
00014    static const Real MOVE_GAIN = 0.005f / ::exp(0.02f);
00015    static const Real ROTATE_GAIN = 0.01f / ::exp(-0.02f);
00016 
00017    /****************************************/
00018    /****************************************/
00019 
00020    void CQTOpenGLCamera::Init(TConfigurationNode& t_tree) {
00021       if(NodeExists(t_tree, "camera")) {
00022          try {
00023             TConfigurationNode tCameraNode;
00024             tCameraNode = GetNode(t_tree, "camera");
00025             TConfigurationNodeIterator itSettingss;
00026             SInt32 nIdx;
00027             for(itSettingss = itSettingss.begin(&tCameraNode);
00028                 itSettingss != itSettingss.end();
00029                 ++itSettingss) {
00030                GetNodeAttribute(*itSettingss, "idx", nIdx);
00031                if(nIdx >=0 && nIdx <= 11) {
00032                   m_sSettings[nIdx].Init(*itSettingss);
00033                }
00034                else {
00035                   THROW_ARGOSEXCEPTION("Error initializing QTOpenGL camera settings: value given for 'idx' is out of bounds. Value = \"" << nIdx << "\", allowed [0-9].");
00036                }
00037             }
00038          }
00039          catch(CARGoSException& ex) {
00040             THROW_ARGOSEXCEPTION_NESTED("Error initializing QTOpenGL camera settings", ex);
00041          }
00042       }
00043    }
00044 
00045    /****************************************/
00046    /****************************************/
00047 
00048    void CQTOpenGLCamera::SSettings::Init(TConfigurationNode& t_tree) {
00049       /* Get positional attributes */
00050       GetNodeAttribute(t_tree, "position", Position);
00051       GetNodeAttribute(t_tree, "look_at", Target);
00052       /* Calculate the Forward vector */
00053       Forward = (Target - Position).Normalize();
00054       /* Was the up vector specified? */
00055       if(!NodeAttributeExists(t_tree, "up")) {
00056          /* Calculate the Left vector
00057             It is located on a plane parallel to XY
00058             It is perpendicular to the projection of Forward on that plane
00059          */
00060          if(Forward.GetX() != 0 || Forward.GetY() != 0) {
00061             CVector2 cLeftXY(Forward.GetX(), Forward.GetY());
00062             cLeftXY.Perpendicularize();
00063             Left.Set(cLeftXY.GetX(), cLeftXY.GetY(), 0.0f);
00064             Left.Normalize();
00065          }
00066          else {
00067             LOGERR << "[WARNING] The given camera position is ambiguous, "
00068                    << "and a standard attitude has been used to calculate it. "
00069                    << "Consider specifying the \"up\" vector in the .argos file "
00070                    << "for more precise placement."
00071                    << std::endl;
00072             Left.Set(0.0f, 1.0f, 0.0f);
00073          }
00074          /* Calculate the Up vector with a cross product */
00075          Up = Forward;
00076          Up.CrossProduct(Left).Normalize();
00077       }
00078       else {
00079          /* Get Up vector */
00080          GetNodeAttribute(t_tree, "up", Up);
00081          Up.Normalize();
00082          /* Calculate the Left vector with a cross product */
00083          Left = Up;
00084          Left.CrossProduct(Forward).Normalize();
00085       }
00086       /* Get optional optics parameters */
00087       Real fValue;
00088       GetNodeAttributeOrDefault<Real>(t_tree, "lens_focal_length", fValue, 20.0f);
00089       LensFocalLength = fValue * 0.001f;
00090       CalculateYFieldOfView();
00091    }
00092 
00093    /****************************************/
00094    /****************************************/
00095 
00096    void CQTOpenGLCamera::SSettings::RotateUpDown(const CRadians& c_angle) {
00097       /* Rotate around the local Y axis (Left)
00098          The rotation matrix corresponding to this rotation is:
00099          
00100          | Fcos(a)-Usin(a)   L   Ucos(a)+Fsin(a) |
00101          
00102          where a is c_angle
00103          L is the Left vector (local Y)
00104          U is the Up vector (local Z)
00105          F is the Forward vector (local X)
00106       */
00107       /* Calculate the new Up vector, given by:
00108          Ucos(a)+Fsin(a)
00109       */
00110       /* Same, but faster than
00111          cNewUp = Up * Cos(c_angle) + Forward * Sin(c_angle);
00112       */
00113       CVector3 cNewUp(Up);
00114       cNewUp *= Cos(c_angle);
00115       cNewUp += Forward * Sin(c_angle);
00116       /* Check whether the rotation exceeds the limits */
00117       if(cNewUp.GetAngleWith(CVector3::Z) > CRadians::PI_OVER_TWO) {
00118          /*
00119           * The rotation exceeds the limits
00120           * The camera Up vector would form an angle bigger than 90 degrees with
00121           * the global Z axis
00122           */
00123          /* We force the Up vector to lie on the XY plane */
00124          Up.SetZ(0.0f);
00125          Up.Normalize();
00126          if(Forward.GetZ() < 0.0f) {
00127             /* Forward was looking down, set it to -Z */
00128             Forward = -CVector3::Z;
00129          }
00130          else {
00131             /* Forward was looking up, set it to Z */
00132             Forward = CVector3::Z;
00133          }
00134       }
00135       else {
00136          /* The rotation is OK */
00137          Up = cNewUp;
00138          Up.Normalize();
00139          /* Now calculate the new Forward vector with a cross product
00140             NOTE: the Left vector, being the rotation axis, remains
00141             unchanged!
00142          */
00143          Forward = Left;
00144          Forward.CrossProduct(Up).Normalize();
00145       }
00146    }
00147 
00148    /****************************************/
00149    /****************************************/
00150 
00151    void CQTOpenGLCamera::SSettings::RotateLeftRight(const CRadians& c_angle) {
00152       /* Rotate around the local Z axis (Up)
00153          The rotation matrix corresponding to this rotation is:
00154          
00155          | Fcos(a)+Lsin(a)   -Fsin(a)+Lcos(a)   U |
00156          
00157          where a is c_angle
00158          L is the Left vector (local Y)
00159          U is the Up vector (local Z)
00160          F is the Forward vector (local X)
00161       */
00162 
00163       /* Calculate the new Forward vector, given by:
00164          Fcos(a)+Lsin(a)
00165       */
00166       /* Same, but faster than
00167          Forward = Forward * Cos(c_angle) + Left * Sin(c_angle);
00168       */
00169       Forward *= Cos(c_angle);
00170       Forward += Left * Sin(c_angle);
00171       Forward.Normalize();
00172       /* Now calculate the new Left vector with a cross product
00173          NOTE: the Up vector, being the rotation axis, remains
00174          unchanged!
00175       */
00176       Left = Up;
00177       Left.CrossProduct(Forward).Normalize();
00178    }
00179 
00180    /****************************************/
00181    /****************************************/
00182 
00183    void CQTOpenGLCamera::SSettings::RotateLeftRight2(const CRadians& c_angle) {
00184       /* This rotation is performed around the global Z axis.
00185          To this aim, along with the global Z axis we project the
00186          Forward and Left axes onto the XY plane and use these as
00187          additional axis to build up the rotation matrix.
00188          With the matrix, we rotate the projected Forward and Left vectors.
00189          We then transform the projected vectors into the final ones.
00190          Finally, we cross-product the two vectors to obtain the new Up vector.
00191       */
00192       if(Forward.GetX() != 0 || Forward.GetY() != 0) {
00193          /* Project Forward onto the XY axis */
00194          CVector3 cForwardXY(Forward.GetX(), Forward.GetY(), 0.0f);
00195          /* Save its length: it will be used to restore the Z coordinate correctly */
00196          Real cForwardXYLength = cForwardXY.Length();
00197          /* Normalize the projection */
00198          cForwardXY.Normalize();
00199          
00200          /* Project Left onto the XY axis and normalize the result */
00201          CVector3 cLeftXY = CVector3::Z;
00202          cLeftXY.CrossProduct(cForwardXY).Normalize();
00203          
00204          /* The rotation matrix corresponding to this rotation is:
00205             
00206             | Fcos(a)+Lsin(a)   -Fsin(a)+Lcos(a)   Z |
00207             
00208             where a is c_angle
00209             L is the Left vector projected on XY
00210             F is the Forward vector projected on XY
00211             Z is the global Z vector
00212          */
00213          
00214          /* Calculate the new cForwardXY vector, given by:
00215             Fcos(a)+Lsin(a)
00216             We keep the unrotated cForwardXY vector, because we will
00217             need it for rotating Left too.
00218          */
00219          /* Same, but faster than
00220             CVector3 cNewForwardXY = cForwardXY * Cos(c_angle) + cLeftXY * Sin(c_angle);
00221          */
00222          CVector3 cNewForwardXY(cForwardXY);
00223          cNewForwardXY *= Cos(c_angle);
00224          cNewForwardXY += cLeftXY * Sin(c_angle);
00225          cNewForwardXY.Normalize();
00226          /* Update Forward from cNewForwardXY: we want to keep the Z coordinate
00227             of Forward unchanged cause we rotated around the global Z, but we
00228             need to update the X and Y coordinates. Right now, cNewForwardXY has
00229             zero Z coordinate and length 1, which means that its X and Y are wrong.
00230             To get the right values, we need to scale it back to the length of the
00231             projection of Forward onto the XY plane. Once this is done, the X and Y
00232             coordinates are right.
00233          */
00234          /* Scale the vector back to the right length */
00235          cNewForwardXY *= cForwardXYLength;
00236          /* Finally, update Forward */
00237          Forward.SetX(cNewForwardXY.GetX());
00238          Forward.SetY(cNewForwardXY.GetY());
00239          Forward.Normalize();
00240          
00241          /* Calculate the new Left vector, given by:
00242             -Fsin(a)+Lcos(a)
00243          */
00244          /* Same, but faster than
00245             Left = cLeftXY * Cos(c_angle) - cForwardXY * Sin(c_angle);
00246          */
00247          Left = cLeftXY;
00248          Left *= Cos(c_angle);
00249          Left -= cForwardXY * Sin(c_angle);
00250          Left.Normalize();
00251          
00252          /* To calculate the new Up vector, a cross-product is enough */
00253          Up = Forward;
00254          Up.CrossProduct(Left).Normalize();
00255       }
00256    }
00257 
00258    /****************************************/
00259    /****************************************/
00260 
00261    void CQTOpenGLCamera::SSettings::Translate(const CVector3& c_delta) {
00262       Position += Forward * c_delta.GetX() + Left * c_delta.GetY() + Up * c_delta.GetZ();
00263       Target = Position;
00264       Target += Forward;
00265    }
00266 
00267    /****************************************/
00268    /****************************************/
00269 
00270    void CQTOpenGLCamera::SSettings::Do() {
00271       gluLookAt(
00272          Position.GetX(),
00273          Position.GetY(),
00274          Position.GetZ(),
00275          Target.GetX(),
00276          Target.GetY(),
00277          Target.GetZ(),
00278          Up.GetX(),
00279          Up.GetY(),
00280          Up.GetZ());
00281    }
00282 
00283    /****************************************/
00284    /****************************************/
00285 
00286    void CQTOpenGLCamera::SSettings::CalculateYFieldOfView() {
00287       YFieldOfView = ToDegrees(2.0f * ATan2(0.027f * 0.5f, LensFocalLength));
00288    }
00289 
00290    /****************************************/
00291    /****************************************/
00292 
00293    void CQTOpenGLCamera::SSettings::CalculateSensitivity() {
00294       MotionSensitivity = MOVE_GAIN * ::exp(LensFocalLength);
00295       RotationSensitivity = ROTATE_GAIN * ::exp(-LensFocalLength);
00296    }
00297 
00298    /****************************************/
00299    /****************************************/
00300 
00301    CQTOpenGLCamera::CQTOpenGLCamera() :
00302       m_unActiveSettings(0) {
00303    }
00304 
00305    /****************************************/
00306    /****************************************/
00307 
00308    CQTOpenGLCamera::~CQTOpenGLCamera() {
00309    }
00310    
00311    /****************************************/
00312    /****************************************/
00313 
00314    void CQTOpenGLCamera::Rotate(const QPoint& c_delta) {
00315       m_sSettings[m_unActiveSettings]
00316          .RotateUpDown(CRadians(m_sSettings[m_unActiveSettings].RotationSensitivity * c_delta.y()));
00317       m_sSettings[m_unActiveSettings]
00318          .RotateLeftRight2(CRadians(-m_sSettings[m_unActiveSettings].RotationSensitivity * c_delta.x()));
00319       m_sSettings[m_unActiveSettings]
00320          .Target = m_sSettings[m_unActiveSettings].Position;
00321       m_sSettings[m_unActiveSettings]
00322          .Target += m_sSettings[m_unActiveSettings].Forward;
00323    }
00324    
00325    /****************************************/
00326    /****************************************/
00327 
00328    void CQTOpenGLCamera::Move(SInt32 n_forwards_backwards,
00329                               SInt32 n_sideways,
00330                               SInt32 n_up_down) {
00331       m_sSettings[m_unActiveSettings].Translate(
00332          CVector3(m_sSettings[m_unActiveSettings].MotionSensitivity * n_forwards_backwards,
00333                   m_sSettings[m_unActiveSettings].MotionSensitivity * n_sideways,
00334                   m_sSettings[m_unActiveSettings].MotionSensitivity * n_up_down));
00335    }
00336 
00337    /****************************************/
00338    /****************************************/
00339 
00340 }
```

---

Generated on 10 Jul 2018 for ARGoS by 
 1.6.1
